# Association Between Smoking and Outcomes of Autoimmune Hepatitis

**DOI:** 10.7759/cureus.66753

**Published:** 2024-08-13

**Authors:** Marah Alarawi, Mona Alfares, Afrah Alzaraija, Haneen Felemban, Lujain Alzahrani, Nada Almadkhali

**Affiliations:** 1 Medical School, King Abdulaziz University Faculty of Medicine, Jeddah, SAU; 2 Infectious Diseases, King Abdulaziz University Hospital, Jeddah, SAU

**Keywords:** hepatitis, autoimmune, outcomes, smoking, cigarette, association

## Abstract

Background

Autoimmune hepatitis (AIH) is a chronic liver disorder caused by the immune system targeting liver cells. The etiology of AIH remains undefined. Therefore, we aim to explore the relationship between cigarette smoking and AIH.

Methods

A retrospective study was done at the Department of Medicine, King Abdulaziz University Hospital (KAUH), Jeddah, Saudi Arabia. Forty-six inpatients and outpatients managed at KAUH from 2016 to 2021 and diagnosed with AIH were included. Data about patients’ age, gender, smoking state, type of liver disease, and any other autoimmune disease were collected.

Results

In all, 10.9% (n = 5) of patients were active smokers, and 60.0% (n = 3) used cigarettes. The median number of cigarettes smoked per day was 17, while 56.5% (n = 26) had a positive family history of smoking, and 41.3% (n = 19) were passive smokers. Of them, 39.1% (n = 18) had at least one complication of AIH, such as liver cirrhosis, which is the most frequent complication (61.1%; n = 11), followed by esophageal varices (22.2%; n = 4), liver fibrosis (5.6%; n = 1), and fatty liver (5.6%; n = 1). The presence of any complication was not associated with patients’ demographics or smoking status. On the other hand, liver cirrhosis was significantly higher among currently active smokers.

Conclusion

No relationship was found between smoking and the AIH outcomes. Future multi-center studies on larger samples are needed.

## Introduction

Autoimmune hepatitis (AIH) is a severe inflammatory liver disease that affects children and adults worldwide. It causes histological, biochemical, and serological changes that result in the autoimmune‐mediated destruction of the liver cells. The etiology of AIH is unclear, but over time, it was seen that genetics, environmental factors, and history of being infected with certain organisms triggered the disease [1,2].

Smoking is a major factor in many pathological conditions and has been linked to the development of various autoimmune diseases. Also, it contains numerous toxic, carcinogenic, stable and unstable free radicals, reactive oxygen species (ROS), and mutagenic chemicals that cause biological oxidative damage. Continuous exposure to these chemicals leads to an immense amount of damage to human health [3,4].

Smoking also has a role in autoimmune diseases by induction of oxidative stress that leads to dysregulating DNA demethylation, upregulation of immune genes, and leading to autoreactivity, which is responsible for the development of autoimmune diseases such as AIH [5-7].

Previous research found that the progression of systemic lupus erythematosus (SLE), rheumatoid arthritis (RA), multiple sclerosis (MS), Graves’ hyperthyroidism, and primary biliary cirrhosis (PBC) was causally related to cigarette smoking [5].

There is no data on the association between AIH and cigarette smoking in Saudi Arabia [8]. As such, understanding factors that might affect the prognosis and quality of life in patients suffering from AIH is essential. To fill this gap, this study aimed to demonstrate the relationship between cigarette smoking and AIH outcomes at King Abdulaziz University Hospital (KAUH), Jeddah, Saudi Arabia.

## Materials and methods

Study design, setting, and population

This study was conducted retrospectively at the Department of Medicine, KAUH, between 2016 and 2021. All participants provided informed consent. It involved both inpatients and outpatients who were diagnosed with AIH. The inclusion criteria were strictly limited to individuals with a confirmed diagnosis of AIH. On the other hand, exclusion criteria encompassed patients diagnosed with cirrhosis not attributed to AIH, as well as those suffering from hepatitis due to non-autoimmune causes. This approach ensured the study focused solely on AIH, thereby enhancing the accuracy and relevance of the findings. By focusing on these specific criteria, the researchers aimed to isolate the effects and characteristics of AIH. The study’s time frame and patient selection criteria were carefully chosen to maximize the validity and applicability of the results.

Data collection

Medical records of 57 patients were reviewed, and the sample size was calculated using a confidence interval of 95% and a margin of error of 6%. We were initially provided with a list of 105 patients who were diagnosed with AIH, of which 20 patients were deceased, 28 patients could not be reached, and 46 patients were contacted. We collected data from 46 patients. However, we excluded data from 11 patients who did not have AIH (three patients had no liver disease, and eight patients had either fatty liver disease, non-AIH, cirrhosis, etc.). Therefore, the records of a total of 46 patients with AIH were analyzed in the current study. A pre-designed data sheet was used to collect data about patients’ age, gender, smoking state, type of liver disease, and any other autoimmune disease.

Data analysis

We used IBM SPSS Statistics, version 26.0 (IBM Corp., Armonk, NY) for data analysis. To present the categorical data, we used frequencies and percentages, whereas numerical variables were expressed as median and interquartile ranges (IQRs). Fisher’s exact test or Pearson’s chi-squared test was used to test for the association between AIH and the factors that cause complications. Fisher’s test was used to determine whether the proportion of the data collected by two or more categorical variables is randomized. The chi-squared test compares the expected results and the observed results and determines whether the difference between them is due to variables that we are studying or by chance. The statistical significance was considered at p < 0.05.

## Results

More than one-third of the patients (39.1%; n = 18) had an age ranging from 18 to <40 years, and less than two-thirds of them (63.0%; n = 29) were females. Five (10.9%) patients were active smokers; of them, three (60.0%) used cigarettes, one (20.0%) used shisha, and one (20.0%) used e-cigarettes. The median (IQR) number of cigarettes smoked per day was 17.0 (13.5, 18.5). Rather than those active smokers, five (10.9%) patients were ex-smokers, and all of them had used cigarettes.

The median time since smoking cessation was 8.5 years (8.2, 8.8). A positive family history of smoking was prevalent among 56.5% (n = 26) of patients, while 41.3% (n = 19) of patients were deemed passive smokers. In this study, non-smokers represented 37.0% (n = 17) of the sample, whereas 63.0% (n = 29) were smokers (either active, passive, or ex-smokers) (Table 1).

**Table 1:** Demographic and smoking-related characteristics IQR, interquartile range

Parameter	Category	N (%)
Age (years)	<18	9 (19.6%)
	18 to <40	18 (39.1%)
	40 to <60	14 (30.4%)
	60 or more	5 (10.9%)
Gender	Male	17 (37.0%)
	Female	29 (63.0%)
Alcohol consumption	Yes	0 (0.0%)
Currently receiving other non-prescribed remedies or over-the-counter medications	Yes	4 (8.7%)
Current smoker	Yes	5 (10.9%)
Type of current smoking	Cigarette	3 (60.0%)
	Shisha	1 (20.0%)
	e-cigarettes	1 (20.0%)
Number of cigarettes smoked per day	N, median (IQR)	17.0 (13.5, 18.5)
Duration of smoking?	Years, median (IQR)	15.0 (5.0, 20.0)
Ex-smoker	Yes	5 (10.9%)
What type do you smoke?	Cigarette	5 (100.0%)
Time since you had ever started smoked	Years, median (IQR)	9.0 (2.8, 15.5)
Time since you quit smoking	Years, median (IQR)	8.5 (8.2, 8.8)
How many cigarettes did you smoke a day?	N, median (IQR)	10.0 (3.0, 20.0)
Family history of smoking	Yes	26 (56.5%)
Where do they smoke?	Indoor	14 (53.8%)
	Outdoor	12 (46.2%)
What do they smoke?	Cigarette	19 (82.6%)
	Shisha	3 (13.0%)
	e-cigarette	1 (4.3%)
How many cigarettes do they smoke a day?	N, median (IQR)	10.0 (3.8, 16.2)
How long have they been smoking?	Years, median (IQR)	15.0 (10.0, 20.0)
Passive smoker	Yes	19 (41.3%)
Smoking status	Smoker	29 (63.0%)
	Non-smoker	17 (37.0%)

Patients with AIH had been diagnosed with their disease primarily via laboratory testing (39.1%; n = 18) and liver biopsy (30.4%; n = 14). Additionally, 39.1% (n = 18) of them had been diagnosed at <18 years of age, and the majority of them (80.4%; n = 37) were diagnosed at KAUH. Almost one-quarter of patients (26.1%; n = 12) had a positive family history of autoimmune hepatitis. Other concomitant autoimmune diseases were prevalent among 19.6% (n = 9) of patients; these included type I diabetes mellitus (44.4%; n = 4), SLE (22.2%; n = 2), RA (11.1%; n = 1), vitiligo (11.1%; n = 1), and celiac disease (11.1%; n = 1), as shown in Table 2.

**Table 2:** Characteristics of autoimmune hepatitis and other concomitant autoimmune diseases IQR, interquartile range; KAUH, King Abdulaziz University Hospital; SLE, systemic lupus erythematosus; T1DM, type 1 diabetes mellitus

Parameter	Category	N (%)
Time since diagnosis (years)	Median (IQR)	6.0 (5.0, 12.5)
At what age were you diagnosed?	<18	18 (39.1%)
	18 to <40	14 (30.4%)
	40 to <60	13 (28.3%)
	60 or more	1 (2.2%)
How were you diagnosed?	Lab testing	18 (39.1%)
	Endoscopy	2 (4.3%)
	Ultrasound	3 (6.5%)
	Liver biopsy	14 (30.4%)
	CT scan	12 (26.1%)
	Symptoms	8 (17.4%)
Where were you diagnosed?	KAUH	37 (80.4%)
	Other hospitals	9 (19.6%)
Is there any other family member diagnosed with a similar diagnosis?	Yes	12 (26.1%)
Other autoimmune disease	Yes	9 (19.6%)
Type of other autoimmune disease	SLE	2 (22.2%)
	T1DM	4 (44.4%)
	Rheumatoid arthritis	1 (11.1%)
	Vitiligo	1 (11.1%)
	Celiac disease	1 (11.1%)

In general, 18 (39.1%) patients had at least one complication of AIH. Liver cirrhosis was the most frequently reported complication (61.1%; n = 11), followed by esophageal varices (22.2%; n = 4), liver fibrosis (5.6%; n = 1), and fatty liver (5.6%; n = 1).

The development of any complications was not associated with patients’ gender, age, or receiving over-the-counter medications. Additionally, the smoking status was not associated with the development of any complications (Tables 3, 4).

**Table 3:** Assessment of factors associated with the incidence of any complication due to autoimmune hepatitis

Parameter	Category	Any complication		
		No, N = 28	Yes, N = 18	p-value
Age (years)	<18	6 (21.4%)	3 (16.7%)	0.707
	18 to <40	11 (39.3%)	7 (38.9%)	
	40 to <60	7 (25.0%)	7 (38.9%)	
	60 or more	4 (14.3%)	1 (5.6%)	
Gender	Male	13 (46.4%)	4 (22.2%)	0.097
	Female	15 (53.6%)	14 (77.8%)	
Currently receiving other non-prescribed remedies or over-the-counter medications	Yes	2 (7.1%)	2 (11.1%)	0.639
Smoking status	Current smoker	1 (3.6%)	4 (22.2%)	0.069
	Ex-smoker	5 (17.9%)	0 (0.0%)	0.140
	Family history of smoking	16 (57.1%)	10 (55.6%)	0.916
	Passive smoker	13 (46.4%)	6 (33.3%)	0.379
	Smoker	19 (67.9%)	10 (55.6%)	0.399

**Table 4:** Assessment of factors associated with the incidence of cirrhosis or esophageal varices as complications due to autoimmune hepatitis

Parameter	Category	Cirrhosis			Esophageal varices		
		No, N = 35	Yes, N = 11	p-value	No, N = 42	Yes, N = 4	p-value
Age (years)	<18	6 (17.1%)	3 (27.3%)	0.371	9 (21.4%)	0 (0.0%)	0.445
	18 to <40	15 (42.9%)	3 (27.3%)		17 (40.5%)	1 (25.0%)	
	40 to <60	9 (25.7%)	5 (45.5%)		12 (28.6%)	2 (50.0%)	
	60 or more	5 (14.3%)	0 (0.0%)		4 (9.5%)	1 (25.0%)	
Gender	Male	14 (40.0%)	3 (27.3%)	0.501	17 (40.5%)	0 (0.0%)	0.281
	Female	21 (60.0%)	8 (72.7%)		25 (59.5%)	4 (100.0%)	
Currently receiving other non-prescribed remedies or over-the-counter medications	Yes	3 (8.6%)	1 (9.1%)	>0.999	2 (4.8%)	2 (50.0%)	0.033
Smoking status	Current smoker	1 (2.9%)	4 (36.4%)	0.009	4 (9.5%)	1 (25.0%)	0.379
	Ex-smoker	5 (14.3%)	0 (0.0%)	0.317	5 (11.9%)	0 (0.0%)	>0.999
	Family history of smoking	20 (57.1%)	6 (54.5%)	>0.999	22 (52.4%)	4 (100.0%)	0.121
	Passive smoker	17 (48.6%)	2 (18.2%)	0.092	16 (38.1%)	3 (75.0%)	0.292
	Smoker	23 (65.7%)	6 (54.5%)	0.722	25 (59.5%)	4 (100.0%)	0.281

Liver cirrhosis development was significantly higher among currently active smokers (36.4% vs 2.9%, p = 0.009). A larger proportion of patients who developed esophageal varices were using over-the-counter medications (50.0% vs 4.8%, p = 0.033), yet the complication was not associated with the smoking variables (Table 4).

## Discussion

This study aimed to assess the relationship between cigarette smoking and AIH outcomes. Research analysis and results indicated that there was no association between cigarette smoking and AIH outcomes.

The current study found that 39.1% (n = 18) of the patients had at least one complication of AIH, with liver cirrhosis being the most common. Furthermore, the presence of any complication was unrelated to the patient’s demographics or smoking status. However, the prevalence of liver cirrhosis was significantly higher among current smokers. The current study’s findings are consistent with previous research, which found that 35% of AIH patients had liver cirrhosis. Furthermore, Wong et al. reported that 33.9% of 183 patients with AIH in their study had liver cirrhosis [9]. This finding was supported by another study examining the epidemiology of AIH in the United States [10].

Although the biological pathway by which cigarette smoking increases the prevalence of autoimmune phenomena is not fully understood, several potential mechanisms have been proposed. Cigarette smoke may cause the release of intracellular antigens via tissue hypoxia or toxin-mediated cellular necrosis, thereby overwhelming the immune system’s scavenging capacity and precipitating an immune reaction in susceptible individuals. Byproducts of cigarette smoking can boost auto-reactive B cells [11]. Cigarette smoke contains a polyphenol-rich glycoprotein isolated from tobacco leaves. It has been shown to stimulate peripheral T-lymphocyte proliferation [12].

Cigarette smoke contains extremely high levels of free radicals. It can also increase the production and activation of endogenous free radicals. These toxins interact with DNA, causing genetic mutations and gene activation, which can lead to autoimmune diseases [13].

Even though liver cirrhosis was seen among active smokers, it is inconclusive that the AIH outcome was due to smoking. In 2021, a prospective cohort study conducted in the USA reported that the correlation between smoking and AIH remains questionable [14].

Several studies have shown an association between cigarette smoking and the development of complications of other autoimmune diseases, but there isn’t any study done on AIH [5-7]. The results also showed that there is no relationship between the development of AIH outcomes and the age, gender, or receiving over-the-counter medication. It was seen among the patients who developed esophageal varices and were taking over-the-counter medication. The important result of the present study was the higher prevalence of liver cirrhosis among currently active smokers (36.4% vs 2.9%, p = 0.009). Previous studies revealed that smoking was significantly associated with the development of non-alcoholic fatty liver disease (NAFLD) and NAFLD-cirrhosis compared to those without [15]. At the same time, other studies found an association between cigarette smoking and the progression of fibrosis in chronic liver diseases [16].

A population cohort study discovered a direct link between smoking and liver cirrhosis. This study discovered that among those who smoked 10 or more grams of tobacco per day, women had a hazard ratio of 2.2 (1.4-3.4) for liver cirrhosis, and men had a hazard ratio of 1.4 (0.9-2.2) for liver cirrhosis after adjusting for alcohol consumption [17]. Tobacco use has been linked to 12% of female liver cirrhosis cases and 6% of male liver cirrhosis cases [17]. After six years, smoking was predictive of liver disease in a sample of 1,290,413 women [18].

A careful literature search found no adequate articles published to address the association between cigarette smoking and outcomes of AIH. Thus, this makes it difficult to compare our results to other study results.

Limitations

A limitation of the present study was the small sample size and the fact that it was a single-center study, which hindered the generalization of the study results.

## Conclusions

This research showed no relationship between cigarette smoking and the development of AIH outcomes. It is challenging to get a full picture or to prove the association between smoking and AIH due to the small population available, and some of them denied having the disease. We might have had a different result if we had conducted this study on a larger population. In addition to the population size, it would be helpful to look into how smoking caused the development of AIH outcomes and if we were able to have before and after data on those who stopped smoking. This will be helpful in studying if any improvements were observed after smoking cessation. In addition to those suggestions to improve the study, we could include other methods to collect data to have a better understanding of the relationship between cigarette smoking and AIH complications.
